# Impact of viral multiplex real-time PCR on management of respiratory tract infection: a retrospective cohort study

**DOI:** 10.1186/s41479-017-0028-z

**Published:** 2017-02-25

**Authors:** Lena M. Mayer, Christian Kahlert, Frank Rassouli, Pietro Vernazza, Werner C. Albrich

**Affiliations:** 1 School of Medicine, University of Basel, Klingelbergstasse 61, 4056 Basel, Switzerland; 2 Children’s Hospital of Eastern Switzerland, Claudiusstrasse 6, 9006 St. Gallen, Switzerland; 3 Clinic for Pulmonology & Sleep Medicine, Kantonsspital St. Gallen, Rorschacherstrasse 95, 9007 St. Gallen, Switzerland; 4 Division of Infectious Diseases & Hospital Epidemiology, Kantonsspital St. Gallen, Rorschacherstrasse 95, 9007 St. Gallen, Switzerland

**Keywords:** Multiplex real-time PCR, Respiratory viruses, Antibiotic management

## Abstract

**Background:**

Significance and clinical utility of multiple virus detection by multiplex real-time polymerase chain reaction (rtPCR) in respiratory tract infection remain unclear.

**Methods:**

This retrospective cohort study analyzed how virus detection affected clinical management. During a 27-month period, clinical and laboratory information was collected from all children and adults in two Swiss tertiary centres whose respiratory samples were tested for respiratory viruses with a 16-plex rtPCR test.

**Results:**

Pathogens were identified in 140 of 254 patients (55%); of those patients, there was ≥1 virus in 91 (65%), ≥ 1 bacterium in 53 (38%), and ≥1 virus and bacterium in 11 (8%). Of 80 patients with viral infection, 59 (74%) received antibiotics. Virus detection was associated with discontinuation of antibiotics in 2 of 20 adults (10%) and 6 of 14 children (43%). Overall 12 adults (34%) and 18 children (67%) were managed correctly without antibiotics after virus detection (*p* = 0.01). When taking biomarkers, radiologic presentations, and antibiotic pre-treatment into account, the impact of rtPCR and appropriateness of therapy for clinically viral infections increased to 100% in children and 62% in adults.

**Conclusions:**

A substantial reduction of unnecessary antibiotic prescriptions seems possible. Appropriate application of rtPCR results in respiratory tract infections should be encouraged.

## Background

Establishing the etiology of respiratory tract infections (RTI) is often difficult due to the lack of a diagnostic gold standard and the inability to detect the causative pathogen. A large proportion of RTI is believed to be caused by respiratory viruses [1–6]. With widespread availability of molecular methods such as multiplex real-time polymerase chain reaction (rtPCR), clinical workflow has changed dramatically and the sensitivity of viral diagnostics has increased remarkably compared to conventional methods [3, 7–13]. However, the significance of virus detection remains unclear as the presence of a virus does not prove causality. Many respiratory viruses can be carried by asymptomatic children [4, 6, 14] and can be shed over prolonged periods, thus discrimination between carriage and infection is challenging [13–15]. Viral infection was reported previously to predispose to bacterial super-infection and concerns about possible bacterial co-infection remain [3, 7–13, 16–19]. Ruling out a bacterial infection with traditional microbiological techniques is frequently impossible [1, 5]*.* The predictive value of clinical signs to differentiate between viral and bacterial infection is also low [13, 17, 20–22]. On these grounds, clinical management of patients after respiratory virus diagnosis is controversial and surprisingly poorly studied. Overall, extensive yet often unnecessary use of antibiotics in RTI is common [1, 5, 6]. This adult and pediatric retrospective cohort study analyzed whether identification of a virus by multiplex rtPCR was associated with changes in the antibiotic treatment.

## Methods

### Study design and population

This was a retrospective cohort study of all pediatric and adult in- and outpatients in whom a 16-plex rtPCR assay for respiratory viruses was performed for upper and lower RTI, from either the Kantonsspital St. Gallen or Children’s hospital of Eastern Switzerland. Both hospitals are tertiary-care Swiss teaching hospitals with active infectious diseases consult services and easily accessible web-based local guidelines (www.guidelines.ch) for the treatment of community-acquired or hospital-acquired pneumonia. The guidelines are strongly recommended for use but not strictly reinforced, and include use of rtPCR as optional diagnostic in hospitalized patients, particularly with immune suppression. The study period was from September 2012 (when this assay was introduced) to November 2014. The documented application dates of anti-infective medication were matched with the date of rtPCR analysis in order to determine whether treatment was changed in response to rtPCR results. The main outcome of interest was whether antibiotic therapy was modified according to results of multiplex rtPCR. Secondary outcomes were prevalence and distribution of positive results of rtPCR, complications, length of stay (LOS), and antibiotic therapy depending on identified pathogens.

### Data collection

To identify patients, the database of the Centre for Laboratory Medicine was searched. Medical records were retrospectively analyzed to obtain basic demographic, clinical, laboratory and radiological parameters and data on clinical management. All chest radiographs (CXR) and computed tomographies (CT) for adults and children were reviewed by a pulmonologist and a pediatrician, respectively.

### Clinical definitions

rtPCR results were available within 24 hours after testing. The application dates of anti-infective medication as documented in the medical records were correlated with the day of, or the day after, rtPCR analysis in order to define whether treatment initiation or discontinuation was associated with the results of viral testing.

The identified pathogens were retrospectively determined as relevant by an infectious disease specialist who integrated all available information. Etiologies were divided in four mutually exclusive groups: (i) bacterial (≥1 bacteria); (ii) viral (≥1 respiratory viruses); (iii) mixed (≥1 bacteria and ≥1 respiratory viruses); or (iv) no pathogen (including fungi, non-respiratory virus, bacterial contaminant including coagulase-negative staphylococci, propionibacterium, corynebacterium, colonizing oral and respiratory flora). For some children, rapid detection tests (Alere BinaxNOW Influenza A&B, Quidel QuickVue RSV Test) for respiratory syncytial virus (RSV) and influenza A/B virus were available. These results were additionally considered in forming the different groups.

For each patient, changes of antibiotic management, i.e. either starting or stopping antibiotics, were determined. All other situations were defined as no change.

Management was considered correct for viral infections if there was no antibiotic treatment before and after rtPCR *or* antibiotics were stopped after positive rtPCR results became available; for bacterial or mixed infections, if antibiotics were given before and after rtPCR *or* were started after a negative rtPCR result. Patients with an indication other than RTI for antibiotic therapy were excluded. For patients with febrile neutropenia without a specific focus, the antibiotic indication was defined as “other” because antibiotics would usually not be stopped despite a viral detection. If a patient underwent repeated testing for respiratory viruses within 3 weeks, only the first positive result was counted. If the time interval was longer or the detected viruses diverged, a different episode was presumed and analyzed separately [23].

To reflect clinical decision making in real-life situations, sub-analyses were performed. Cases without detection of bacteria (i.e. patients with viral etiology or with no pathogen) were evaluated as clinically bacterial if they fulfilled one of the following criteria: unilobar or multilobar pulmonary infiltrate on CT or CXR; C-reactive protein (CRP) >100 mg/l; procalcitonin (PCT) >0.25 μg/l; antibiotic therapy before rtPCR *and* with indeterminate biomarkers (CRP >100 mg/l *and* PCT ≤0.25 μg/l *or* CRP 51–100 mg/l *and* PCT not available). These criteria were adapted from earlier publications [17, 21] regarding the diagnosis of bacterial lower RTI.

The subgroup of patients with a viral etiology who had no clinically bacterial infection was considered to have a clinically viral infection. Children (<18 years) and adults (≥18 years) were analyzed separately. Sepsis was defined according to standard criteria at the time [24]. Systemic inflammatory response syndrome (SIRS) criteria for children were defined as age-specific [25].

For the imaging, if patients had interstitial infiltrates or ground glass opacities in the absence of unilobar or multilobar infiltrates, only therapy against “atypical” pathogens (macrolide, quinolone, tetracycline; but not antibiotics for coverage of “typical” bacterial pathogens) was considered appropriate. If both CXR and CT were available, only CT readings were used for further analyses. An infiltrate was required for the diagnosis of pneumonia but no radiography was needed to diagnose a RTI in general.

### Laboratory procedures

The multiplex rtPCR was performed according to the manufacturer’s instruction (Seegene, Korea). The Anyplex™ II RV16 detection kit (Seegene, Korea) detects the following viruses: adenovirus, influenza A virus, influenza B virus, parainfluenza virus 1, parainfluenza virus 2, parainfluenza virus 3, parainfluenza virus 4, rhinovirus A/B/C, RSV A, RSV B, bocavirus 1/2/3/4, human metapneumovirus, coronavirus 229E, coronavirus NL63, coronavirus OC43, and enterovirus. Specimens that arrived before mid-morning from Monday to Friday were processed daily and results were provided by mid-afternoon. Specimens that arrived afterwards were processed on the following workday and reported by mid-afternoon. Dates of specimen collection and testing were available, but not date and time of reporting of results. For CRP, white blood cell count (WBC) and platelets, the most pathologic values within 3 days before and after rtPCR results were documented.

### Statistical analyses

Quantitative variables are described as means ± standard deviations or median and interquartile range (IQR), as appropriate. Qualitative variables are presented as absolute counts and relative percentages. χ2-test or Fisher’s exact test were used to compare proportions, as appropriate. For continuous variables, the 2-sample independent t-test or the Mann-Whitney-U-test were used. *P*-values ≤0.05 (2-sided) were considered statistically significant. Multivariate logistic regression was used to examine whether different predictors were associated with virus detection. Variables with a *p*-value ≤0.05 in the univariate logistic regression were included. SPSS version 20.0 for Windows software, OpenEpi (www.openepi.com) and Microsoft Excel 2010 were used for statistical analyses.

## Results

rtPCR for respiratory viruses was performed on 328 respiratory specimens, of which 74 samples were excluded due to insufficient clinical information or repeated testing of separate specimens for respiratory viruses in the same patient within 3 weeks. Data on 254 patients were analyzed, including 11 sputa, 47 nasopharyngeal swabs, 63 nasopharyngeal aspirates, 123 bronchoalveolar lavages, 9 tracheal aspirates and 1 pleural effusion.

### Clinical characteristics

Baseline characteristics are presented in Tables 1, 2 and 3. One hundred and seven patients (42%) were female, and 72 were children (28%). Mean age in the group with viral infections was lower than in the remaining patients (29 vs. 47 years; *p* < 0.001) but the difference was not significant if children and adults were analyzed separately (children 4.3 vs. 5.7 years; *p* = 0.28; adults 53.6 vs. 56.6 years; *p* = 0.31). There were more patients with neutropenia, hematological malignancy or collagen vascular disease/vasculitis in the viral group (*p* < 0.001; *p* = 0.04; *p* = 0.01). Diagnosis of bronchitis and upper RTI were more common in the viral group (*p* = 0.02; *p* = 0.004).

**Table 1:** Baseline characteristics for adult patients

	*Missing*	*Total*	*Viral* ^*a*^	*Any virus* ^*b*^	*Other* ^c^	*p-value* ^*d*^	
	*values*	*(n = 182)*	*(n = 40)*	*(n = 46)*	*(n = 142)*	*Viral vs. any virus*	*Viral vs. other*
Demographics							
Age, mean years ± SD (range)		55.9 ± 16.1 (18–88)	53.6 ± 16.1 (18–83)	53.9 ± 15.9 (18–83)	56.6 ± 16.1 (18–88)	0.93	0.30
Female sex, n (%)		76 (41.8)	14 (35.0)	19 (41.3)	62 (43.7)	0.55	0.33
Outpatients, n (%)		20 (11.0)	5 (12.5)	5 (10.9)	15 (10.6)	1.00	0.92
Comorbidities, n (%)							
Asthma		13 (7.1)	2 (5.0)	4 (8.7)	11 (7.7)	0.81	0.85
COPD		27 (10.6)	5 (12.5)	7 (15.2)	22 (15.5)	0.72	0.64
Other chronic lung disease^e^		26 (14.3)	2 (5.0)	2 (4.3)	24 (16.9)	1.00	0.06
Solid cancer^f^		25 (13.7)	3 (7.5)	3 (6.5)	22 (15.5)	1.00	0.20
Hematologic malignancy		38 (20.9)	13 (32.5)	15 (32.6)	25 (17.6)	0.99	0.04
Organ transplantation		13 (7.1)	3 (7.5)	3 (6.5)	10 (7.0)	1.00	1.00
Neutropenia		20 (11.0)	9 (22.5)	9 (19.6)	11 (7.7)	0.74	0.03
HIV infection		6 (3.3)	1 (2.5)	2 (4.3)	5 (3.5)	1.00	1.00
Diabetes mellitus		27 (14.8)	6 (15.0)	7 (15.2)	21 (14.8)	0.98	0.97
Collagen vascular disease/Vasculitis		33 (18.1)	4 (10.0)	5 (10.9)	29 (20.4)	1.00	0.13
Systemic steroids		49 (26.9)	11 (27.5)	14 (30.4)	38 (26.8)	0.77	0.93
Other Immunosuppression^g^		21 (11.5)	5 (12.5)	6 (13.0)	16 (11.3)	0.94	1.00
Chronic renal failure		45 (24.7)	11 (27.5)	13 (28.3)	34 (23.9)	0.94	0.65
Clinical findings							
Systolic blood pressure, mmHg, mean ± SD (range)	9	125 ± 24 (63–207)	129 ± 27 (63–207)	128 ± 28 (63–207)	123 ± 22 (67–197)	0.87	0.17
Heart rate, beats/min, mean ± SD (range)	8	94 ± 20 (51–155)	91 ± 19 (51–150)	94 ± 19 (51–150)	94 ± 20 (56–155)	0.47	0.40
Respiratory rate, breaths/min, mean ± SD (range)	130	26 ± 9 (10–60)	23 ± 8 (12–36)	27 ± 13 (12–60)	27 ± 10 (10–60)	0.39	0.23
Body temperature, °C, mean ± SD (range)	20	37.6 ± 1.0 (35.4–42.0)	37.5 ± 0.9 (35.6–39.6)	37.5 ± 0.9 (35.6–39.6)	37.6 ± 1.0 (35.4–42.0)	1.00	0.59
Laboratory findings^h^, mean ± SD (range)							
C-reactive protein (maximum), mg/l	13	151 ± 127 (1–500)	159 ± 148 (1–500)	166 ± 152 (1–500)	148 ± 121 (1–499)	0.83	0.64
White blood cells (maximum), G/l	11	11.5 ± 10.2 (0.0–97.0)	12.2 ± 16.8 (1.8–97.0)	12.6 ± 16.3 (1.8–97.0)	11.3 ± 7.3 (0.0–41.1)	0.91	0.75
Platelets (minimum), G/l	11	202 ± 128 (4–713)	159 ± 117 (5–513)	165 ± 117 (5–513)	215 ± 129 (4–713)	0.82	0.02
Discharge diagnosis, n (%)							
Bronchitis		22 (12.1)	7 (17.5)	7 (15.2)	15 (10.6)	0.78	0.36
Acute exacerbation of COPD		5 (2.7)	1 (2.5)	1 (2.2)	4 (2.8)	1.00	1.00
Upper respiratory tract infection		8 (4.4)	4 (10.0)	4 (8.7)	4 (2.8)	1.00	0.14
Respiratory tract infection, unspecified		7 (3.8)	2 (5.0)	4 (8.7)	5 (3.5)	0.81	0.96
Community-acquired pneumonia		65 (35.7)	21 (52.5)	25 (54.3)	44 (31.0)	0.86	0.01
Hospital-acquired pneumonia		12 (6.6)	0 (0.0)	0 (0.0)	12 (8.5)	^n/a^	0.09
Aspiration pneumonia		1 (0.5)	0 (0.0)	0 (0.0)	1 (0.7)	^n/a^	1.00
Tuberculosis		3 (1.6)	0 (0.0)	0 (0.0)	3 (2.1)	^n/a^	0.95
Other^i^		59 (32.4)	5 (12.5)	5 (10.9)	54 (38.0)	1.00	0.002

°C, degree Celsius, *COPD* chronic obstructive pulmonary disease, *HIV* human immunodeficiency virus, *n* number, *SD* standard deviation

^a^Cases in which only one or more respiratory viruses were detected

^b^Cases in which one or more respiratory viruses were detected (as single or mixed infection)

^c^Includes bacterial etiology, mixed etiology, no pathogen

^d^For continuous variables, 2-sample independent t-test was used. For categorical variables, Mantel-Haenszel chi square or Fisher exact test were used

^e^Other chronic lung diseases, e.g. cystic fibrosis, pulmonary sarcoidosis, pulmonary hypertension

^f^All solid tumors including bronchial carcinoma

^g^Patients with one of the following conditions: primary or secondary antibody deficiency, congenital immunodeficiency, immunosuppressive therapy other than steroids, severe malnutrition with cachexia

^h^Highest/lowest value within a time period of 3 days before and 3 days after date of rtPCR

^i^Other infections (*n* = 10); neoplastic diseases (*n* =6); collagen vascular; other rheumatologic or autoimmune (*n* = 14); sarcoidosis (*n* = 3); non-infectious non-neoplastic pulmonary diseases (*n* = 23); cardiovascular diseases (*n* = 3)

**Table 2:** Baseline characteristics for pediatric patients

	*Missing*	*Total*	*Viral* ^*a*^	*Any virus* ^*b*^	*Other* ^c^	*p-value* ^*d*^	
	*values*	*(n = 72)*	*(n = 40)*	*(n = 45)*	*(n = 32)*	*Viral vs. any virus*	*Viral vs. other*
Demographics							
Age, mean years ± SD (range)		4.9 ± 5.7 (0–17)	4.3 ± 5.4 (0–16)	4.7 ± 5.5 (0–16)	5.7 ± 6.0 (0–17)	0.74	0.30
Female sex, n (%)		31 (43.1)	14 (35.0)	18 (40.0)	17 (53.1)	0.64	0.13
Outpatients, n (%)		3 (4.2)	2 (5.0)	2 (4.4)	1 (3.1)	1.00	1.00
Comorbidities, n (%)							
Asthma		1 (1.4)	1 (2.5)	1 (2.2)	0 (0.0)	1.00	1.00
Other chronic lung disease^e^		13 (18.1)	7 (17.5)	8 (17.8)	6 (18.8)	0.97	0.89
Solid cancer^f^		1 (1.4)	0 (0.0)	0 (0.0)	1 (3.1)	^n/a^	0.89
Haematologic malignancy		10 (13.9)	8 (20.0)	9 (20.0)	2 (6.3)	1.00	0.18
Organ transplantation		1 (1.4)	1 (2.5)	1 (2.2)	0 (0.0)	1.00	1.00
Neutropenia		9 (12.5)	8 (20.0)	8 (17.8)	1 (3.1)	0.79	0.06
Systemic steroids		8 (11.1)	5 (12.5)	6 (13.3)	3 (9.4)	0.91	0.98
Other Immunosuppression^g^		3 (4.2)	3 (7.5)	3 (6.7)	0 (0.0)	1.00	0.33
Clinical findings							
Systolic blood pressure, mmHg, mean ± SD (range)	29	100 ± 18 (59–140)	106 ± 15 (71–140)	105 ± 14 (71–140)	95 ± 18 (59–120)	0.82	0.04
Heart rate, beats/min, mean ± SD (range)	3	136 ± 33 (60–234)	138 ± 32 (60–186)	134 ± 32 (60–186)	135 ± 35 (72–234)	0.58	0.71
Respiratory rate, breaths/min, mean ± SD (range)	15	41 ± 20 (16–103)	41 ± 21 (18–103)	40 ± 21 (16–103)	40 ± 19 (16–88)	0.84	0.86
Body temperature, °C, mean ± SD (range)	3	37.6 ± 1.1 (35.0–40.1)	37.5 ± 1.1 (35.0–39.5)	37.5 ± 1.1 (35.0–39.5)	37.7 ± 1.1 (35.1–40.1)	1.00	0.46
Laboratory findings^h^, mean ± SD (range)							
C-reactive protein (maximum), mg/l	2	85 ± 141 (5–999)	62 ± 77 (5–291)	67 ± 81 (5–293)	113 ± 191 (7–999)	0.78	0.16
White blood cells (maximum), G/l	2	16.0 ± 12.1 (0.2–63.1)	12.6 ± 10.0 (0.2–57.0)	13.6 ± 11.3 (0.2–57.0)	20.1 ± 13.3 (1.8–63.1)	0.68	0.01
Platelets (minimum), G/l	2	246 ± 144 (15–674)	257 ± 175 (15–674)	244 ± 169 (15–674)	234 ± 96 (16–488)	0.73	0.49
Discharge diagnosis, n (%)							
Bronchitis		10 (13.9)	9 (22.5)	9 (20.0)	1 (3.1)	0.78	0.04
Upper respiratory tract infection		14 (19.4)	9 (22.5)	10 (22.2)	5 (15.6)	0.98	0.47
Respiratory tract infection, unspecified		4 (5.6)	4 (10.0)	4 (8.9)	0 (0.0)	1.00	0.18
Community-acquired pneumonia		15 (20.8)	6 (15.0)	8 (17.8)	9 (28.1)	0.73	0.18
Hospital-acquired pneumonia		10 (13.9)	5 (12.5)	6 (13.3)	5 (15.6)	0.91	0.96
Aspiration pneumonia		2 (2.8)	1 (2.5)	2 (4.4)	1 (3.1)	1.00	1.00
Other^i^		17 (23.6)	6 (15.0)	6 (13.3)	11 (34.4)	0.83	0.06

°C, degree Celsius, *COPD* chronic obstructive pulmonary disease, *HIV* human immunodeficiency virus, *n* number, *SD* standard deviation

^a^Cases in which only one or more respiratory viruses were detected

^b^Cases in which one or more respiratory viruses were detected (as single or mixed infection)

^c^Includes bacterial etiology, mixed etiology, no pathogen

^d^For continuous variables, 2-sample independent t-test was used. For categorical variables, Mantel-Haenszel chi square or Fisher exact test were used

^e^Other chronic lung diseases, e.g. cystic fibrosis, pulmonary sarcoidosis, pulmonary hypertension

^f^All solid tumors including bronchial carcinoma

^g^Patients with one of the following conditions: primary or secondary antibody deficiency, congenital immunodeficiency, immunosuppressive therapy other than steroids, severe malnutrition with cachexia

^h^Highest/lowest value within a time period of 3 days before and 3 days after date of rtPCR

^i^Other infections (*n* = 14); neoplastic diseases (*n* = 1); collagen vascular; other rheumatologic or autoimmune (*n* = 1); gastroesophageal diseases (*n* = 1)

**Table 3:** Baseline characteristics for all patients

	*Missing*	*Total*	*Viral* ^*a*^	*Any virus* ^*b*^	*Other* ^c^	*p-value* ^*d*^	
	*values*	*(n = 254)*	*(n = 80)*	*(n = 91)*	*(n = 174)*	*Viral vs. any virus*	*Viral vs. other*
Demographics							
Age, mean years ± SD (range)		41.5 ± 26.9 (0–88)	29.0 ± 27.5 (0–83)	29.6 ± 27.4 (0–83)	47.2 ± 24.7 (0–88)	0.89	<0.001
Female sex, n (%)		107 (42.1)	28 (35.0)	37 (40.7)	79 (45.4)	0.45	0.12
Children, n (%)		72 (28.3)	40 (50.0)	45 (49.5)	32 (18.4)	0.94	<0.001
Outpatients, n (%)		23 (9.1)	7 (8.8)	7 (7.7)	16 (9.2)	0.80	0.91
Comorbidities, n (%)							
Asthma		14 (5.5)	3 (3.8)	5 (5.5)	11 (6.3)	0.87	0.61
COPD		27 (10.6)	5 (6.3)	7 (7.7)	22 (12.6)	0.71	0.13
Other chronic lung disease^e^		39 (15.4)	9 (11.3)	10 (11.0)	30 (17.2)	0.96	0.22
Solid cancer^f^		26 (10.2)	3 (3.8)	3 (3.3)	23 (13.2)	1.00	0.02
Haematologic malignancy		48 (18.9)	21 (26.3)	24 (26.4)	27 (15.5)	0.99	0.04
Organ transplantation		14 (5.5)	4 (5.0)	4 (4.4)	10 (5.7)	1.00	1.00
Neutropenia		29 (11.4)	17 (21.3)	17 (18.7)	12 (6.9)	0.68	<0.001
HIV infection		6 (2.4)	1 (1.3)	2 (2.2)	5 (2.9)	1.00	0.77
Diabetes mellitus		27 (10.6)	6 (7.5)	7 (7.7)	21 (12.1)	0.96	0.28
Collagen vascular disease/Vasculitis		33 (13.0)	4 (5.0)	5 (5.5)	29 (16.7)	1.00	0.01
Systemic steroids		57 (22.4)	16 (20.0)	20 (22.0)	41 (23.6)	0.75	0.53
Other Immunosuppression^g^		24 (9.4)	8 (10.0)	9 (9.9)	16 (9.2)	0.98	0.84
Chronic renal failure		45 (17.7)	11 (13.8)	13 (14.3)	34 (19.5)	0.92	0.26
Clinical findings							
Systolic blood pressure, mmHg, mean ± SD (range)	38	120 ± 25 (59–207)	121 ± 26 (63–207)	119 ± 26 (63–207)	119 ± 24 (59–197)	0.67	0.60
Heart rate, beats/min, mean ± SD (range)	11	106 ± 31 (51–234)	114 ± 35 (51–186)	114 ± 33 (51–186)	102 ± 28 (56–234)	1.00	0.01
Respiratory rate, breaths/min, mean ± SD (range)	145	34 ± 18 (10–103)	37 ± 21 (12–103)	37 ± 20 (12–103)	32 ± 15 (10–88)	1.00	0.17
Body temperature, °C, mean ± SD (range)	23	37.6 ± 1.0 (35.0–42.0)	37.5 ± 1.0 (35.0–39.6)	37.5 ± 1.0 (35.0–39.6)	37.6 ± 1.0 (35.1–42.0)	1.00	0.48
Laboratory findings^h^, mean ± SD (range)							
C-reactive protein (maximum), mg/l	15	132 ± 134 (1–999)	111 ± 127 (1–500)	117 ± 132 (1–500)	141 ± 137 (1–999)	0.77	0.11
White blood cells (maximum), G/l	13	12.8 ± 11.0 (0.0–97.0)	12.4 ± 13.8 (0.2–97.0)	13.1 ± 14.0 (0.2–97.0)	13.1 ± 9.4 (0.0–63.1)	0.75	0.69
Platelets (minimum), G/l	13	215 ± 134 (4–713)	208 ± 156 (5–674)	204 ± 149 (5–674)	218 ± 123 (4–713)	0.87	0.62
Discharge diagnosis, n (%)							
Bronchitis		32 (12.6)	16 (20.0)	16 (17.6)	16 (9.2)	0.69	0.02
Acute exacerbation of COPD		5 (2.0)	1 (1.3)	1 (1.1)	4 (2.3)	1.00	0.99
Upper respiratory tract infection		22 (8.7)	13 (16.3)	14 (15.4)	9 (5.2)	0.88	0.004
Respiratory tract infection, unspecified		11 (4.3)	6 (7.5)	8 (8.8)	5 (2.9)	0.76	0.18
Community-acquired pneumonia		80 (31.5)	27 (33.8)	33 (36.3)	53 (30.5)	0.73	0.60
Hospital-acquired pneumonia		22 (8.7)	5 (6.3)	6 (6.6)	17 (9.8)	0.93	0.36
Aspiration pneumonia		3 (1.2)	1 (1.3)	2 (2.2)	2 (1.1)	1.00	1.00
Tuberculosis		3 (1.2)	0 (0.0)	0 (0.0)	3 (1.7)	n/a	0.64
Other^i^		76 (29.9)	11 (13.8)	11 (12.1)	65 (37.4)	0.75	<0.001

°C, degree Celsius, *COPD* chronic obstructive pulmonary disease, *HIV* human immunodeficiency virus, *n* number, *SD* standard deviation

^a^Cases in which only one or more respiratory viruses were detected

^b^Cases in which one or more respiratory viruses were detected (as single or mixed infection)

^c^Includes bacterial etiology, mixed etiology, no pathogen

^d^For continuous variables, 2-sample independent t-test was used. For categorical variables, Mantel-Haenszel chi square or Fisher exact test were used

^e^Other chronic lung diseases, e.g. cystic fibrosis, pulmonary sarcoidosis, pulmonary hypertension

^f^All solid tumors including bronchial carcinoma

^g^Patients with one of the following conditions: primary or secondary antibody deficiency, congenital immunodeficiency, immunosuppressive therapy other than steroids, severe malnutrition with cachexia

^h^Highest/lowest value within a time period of 3 days before and 3 days after date of rtPCR

^i^Other infections (*n* = 24); neoplastic diseases (*n* = 7); collagen vascular; other rheumatologic or autoimmune (*n* = 15); sarcoidosis (*n* = 3); non-infectious non-neoplastic pulmonary diseases (*n* = 23); cardiovascular diseases (*n* = 3); gastroesophageal diseases (*n* = 1)

### Pathogen identification

**Fig. 1:**
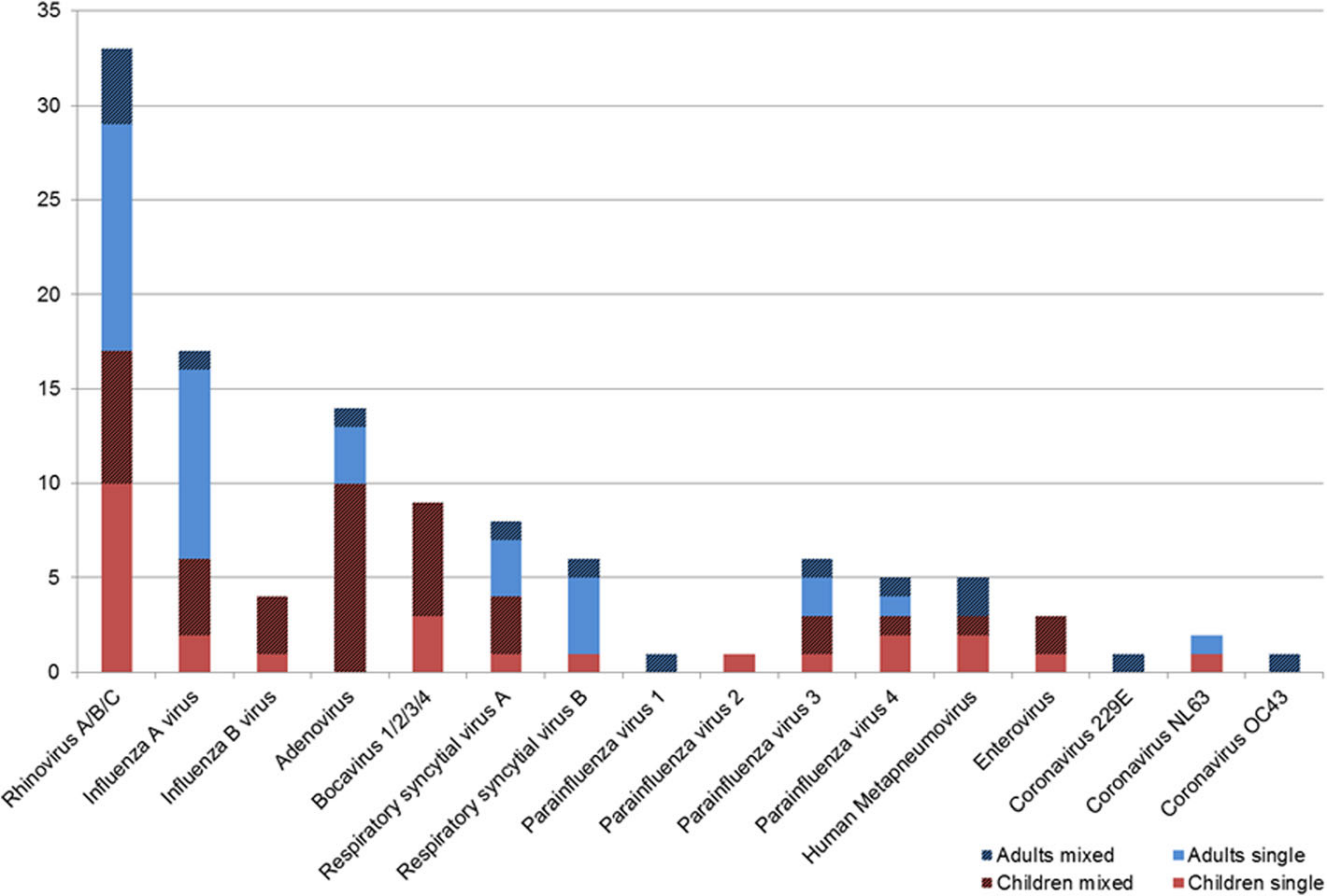
Distribution of respiratory viruses detected as single or mixed pathogen

**Fig. 2:**
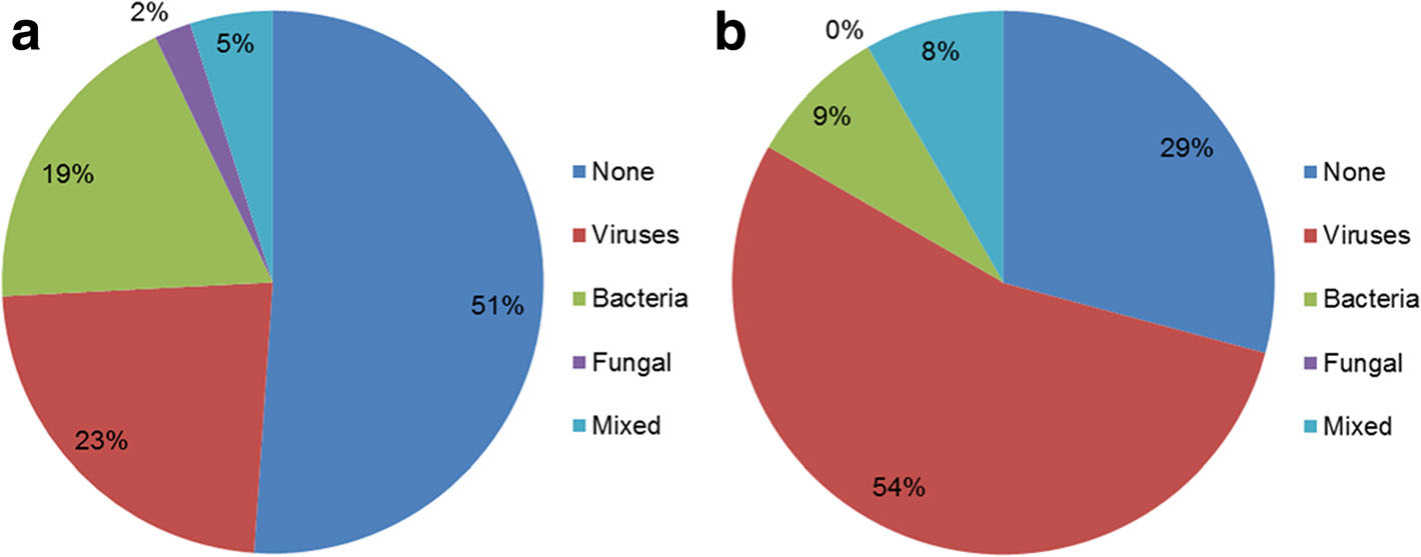
**a** Distribution of identified pathogens for adults. **b** Distribution of identified pathogens for children

Any pathogen was identified in 140 patients (55%), in 89 of 182 adults (49%) and in 51 of 72 children (71%). Among these patients, one or more respiratory virus was detected in 91 (65%), one or more bacteria in 53 (38%) and a mixed viral-bacterial infection in 11 (8%) patients. 45 of 72 children (63%) were infected with one or more respiratory viruses. Compared to children, viral detection in adults was less frequent (25%, *n* = 46; *p* < 0.001). A single pathogen was identified in 99 patients (39%); multiple pathogens were detected in 41 patients (16%). Distribution of detected pathogens and the differences between children and adults are shown in Table 4, and Figs. 1, 2a and b.

**Table 4:** Relevant pathogens identified from respiratory specimens

	*Total (n = 254)*	*Adults (n = 182)*	*Children (n = 72)*
Bacteria, n (%)	66 (26.0)	54 (29.7)	12 (16.7)
*Haemophilus influenzae*	12 (4.7)	11 (6.0)	1 (1.4)
*Klebsiella pneumoniae*	8 (3.1)	6 (3.3)	2 (2.8)
*Staphylococcus aureus*	4 (1.6)	1 (0.5)	3 (4.2)
*Escherichia coli*	4 (1.6)	3 (1.6)	1 (1.4)
*Pseudomonas aeruginosa*	4 (1.6)	4 (2.2)	0 (0.0)
*Streptococcus pneumoniae*	3 (1.2)	2 (1.1)	1 (1.4)
*Streptococcus pyogenes*	3 (1.2)	2 (1.1)	1 (1.4)
*Legionella pneumophila*	3 (1.2)	3 (1.6)	0 (0.0)
*Mycobacterium tuberculosis*	3 (1.2)	3 (1.6)	0 (0.0)
*Mycoplasma pneumoniae*	1 (0.4)	0 (0.0)	1 (1.4)
Other bacteria	21 (8.3)	19 (10.4)	2 (2.8)
Viruses, n (%)	119 (46.9)	54 (29.7)	65 (90.3)
Rhinovirus A/B/C	33 (13.0)	16 (8.8)	17 (23.6)
Influenza A virus	17 (6.7)	11 (6.0)	6 (8.3)
Influenza B virus	4 (1.6)	0 (0.0)	4 (5.6)
Adenovirus	14 (5.5)	4 (2.2)	10 (13.9)
Bocavirus 1/2/3/4	9 (3.5)	0 (0.0)	9 (12.5)
Respiratory syncytial virus A	8 (3.1)	4 (2.2)	4 (5.6)
Respiratory syncytial virus B	6 (2.4)	5 (2.7)	1 (1.4)
Parainfluenza virus 1	1 (0.4)	1 (0.5)	0 (0.0)
Parainfluenza virus 2	1 (0.4)	0 (0.0)	1 (1.4)
Parainfluenza virus 3	6 (2.4)	3 (1.6)	3 (4.2)
Parainfluenza virus 4	5 (2.0)	2 (1.1)	3 (4.2)
Human Metapneumovirus	5 (2.0)	2 (1.1)	3 (4.2)
Enterovirus	3 (1.2)	0 (0.0)	3 (4.2)
Coronavirus 229E	1 (0.4)	1 (0.5)	0 (0.0)
Coronavirus NL63	2 (0.8)	1 (0.5)	1 (1.4)
Coronavirus OC43	1 (0.4)	1 (0.5)	0 (0.0)
Non respiratory viruses	3 (1.2)	3 (1.6)	0 (0.0)
Fungi, n (%)	10 (3.9)	9 (4.9)	1 (1.4)
Pneumocystis jirovecii	6 (2.4)	6 (3.3)	0 (0.0)
Other fungi	4 (1.6)	3 (1.6)	1 (1.4)

Pathogens associated with mixed infections were counted individually

### Antibiotic therapy

Of the 254 patients in the cohort, 61 (24%) received antibiotics before hospitalization, and 149 (59%) of all in- and outpatients received antibiotics at the time of rtPCR testing. Of 80 patients with a viral etiology, 59 (74%) received antibiotics at any time point, 28 of 40 children (70%) and 31 of 40 adults (78%). Inpatients with a bacterial etiology were treated with antibiotics in 92% (*p* = 0.09) and longer than those with a viral etiology or no pathogen (*p* = 0.02; *p* = 0.01). If children and adults were analyzed individually, similar but non-significant trends were observed (Tables 5, 6 and 7).

**Table 5:** Outcome depending on relevant detected pathogen for adult patients

	*Viral*	*Bacterial*	*Mixed*	*No pathogen*	*p-value* ^*a*^		
	*(n = 40)*	*(n = 36)*	*(n = 6)*	*(n = 100)*	*Viral vs. bacterial*	*Viral vs. mixed*	*Viral vs. no pathogen*
LOS inpatients, median days (IQR)	8 (6–21)	21 (13–35)	11.5 (5–44.25)	15 (8–23.5)	<0.001	0.67	0.03
Complications, n (%)							
ICU admission	11 (27.5)	13 (36.1)	3 (50.0)	24 (24.0)	0.42	0.51	0.67
Mechanical ventilation	6 (15.0)	11 (30.6)	3 (50.0)	19 (19.0)	0.11	0.16	0.58
ARDS	3 (7.5)	5 (13.9)	2 (33.3)	6 (6.0)	0.60	0.24	1.00
Sepsis	24 (60.0)	24 (66.7)	5 (83.3)	6 (6.0)^b^	0.55	0.53	n/a
Mortality (all cause)	4 (10.0)	4 (11.1)	1 (16.7)	6 (6.0)	1.00	1.00	0.62
Antibiotic use (any indication)							
Any inpatient antibiotics, n (%)	30/35 (85.7)	29/32 (90.6)	4/6 (66.7)	72/89 (80.9)	0.81	0.54	0.53
Duration of inpatient use, mean days ± SD (range)	12.5 ± 14.3 (0–63)	18.1 ± 16.0 (0–72)	10.3 ± 12.1 (0–31)	10.8 ± 11.4 (0–63)	0.14	0.73	0.49
Discharged receiving oral antibiotics, n (%)	11 (30.6)^c^	17 (53.1)^d^	1 (20.0)^e^	23 (24.5)^f^	0.06	1.00	0.48

*ARDS* acute respiratory distress syndrome, *ICU* intensive care unit, *IQR* interquartile range, *LOS* length of stay, *n* number, *SD* standard deviation

^a^For continuous variables, 2-sample independent t-test or Mann-Whitney-U-test were used. For categorical variables, Mantel-Haenszel chi square or Fisher exact test were used

^b^Sepsis requires a pathogen per definition. In these six cases sepsis was exceptionally defined according to discharge papers

Missing values due to death or ongoing hospitalisation at time of analysis (number): c4; d4; e1; f6. For calculation of percentage and *p*-value missing values were excluded

**Table 6:** Outcome depending on relevant detected pathogen for pediatric patients

	*Viral*	*Bacterial*	*Mixed*	*No pathogen*	*p-value* ^*a*^		
	*(n = 40)*	*(n = 6)*	*(n = 5)*	*(n = 21)*	*Viral vs. bacterial*	*Viral vs. mixed*	*Viral vs. no pathogen*
LOS inpatients, median days (IQR)	18.5 (6–48.75)	47.5 (14–95.75)	34 (17.5–201.5)	37 (8–70)	0.19	0.10	0.45
Complications, n (%)							
ICU admission	18 (45.0)	4 (66.7)	5 (100.0)	12 (57.1)	0.58	0.06	0.37
Mechanical ventilation	8 (20.0)	4 (66.7)	4 (80.0)	11 (52.4)	0.07	0.03	0.01
ARDS	1 (2.5)	0 (0.0)	1 (20.0)	0 (0.0)	1.00	0.42	1.00
Sepsis	17 (42.5)	4 (66.7)	2 (40.0)	0 (0.0)	0.50	1.00	n/a
Mortality (all cause)	2 (5.0)	0 (0.0)	0 (0.0)	0 (0.0)	1.00	1.00	0.85
Antibiotic use (any indication)							
Any inpatient antibiotics, n (%)	28/38 (73.7)	6/6 (100.0)	5/5 (100.0)	18/20 (90.0)	0.38	0.49	0.26
Duration of inpatient use, mean days ± SD (range)	8.6 ± 13.4 (0–73)	14.2 ± 11.0 (0–34)	13.8 ± 7.8 (7–24)	6.2 ± 4.8 (0–18)	0.34	0.40	0.33
Discharged receiving oral antibiotics, n (%)	6 (15.8)^b^	0 (0.0)	1 (20.0)	3 (15.0)^c^	0.78	1.00	1.00

*ARDS* acute respiratory distress syndrome, *ICU* intensive care unit, *IQR* interquartile range, *LOS* length of stay, *n* number, *SD* standard deviation

^a^For continuous variables, 2-sample independent t-test or Mann-Whitney-U-test were used. For categorical variables, Mantel-Haenszel chi square or Fisher exact test were used

Missing values due to death or ongoing hospitalisation at time of analysis (number): b2; c1. For calculation of percentage and *p*-value missing values were excluded

**Table 7:** Outcome depending on relevant detected pathogen for all patients

	*Viral*	*Bacterial*	*Mixed*	*No pathogen*	*p-value* ^*a*^		
	*(n = 80)*	*(n = 42)*	*(n = 11)*	*(n = 121)*	*Viral vs. bacterial*	*Viral vs. mixed*	*Viral vs. no pathogen*
LOS inpatients, median days (IQR)	12 (6–25.5)	22 (13–42.5)	27 (8–72)	16 (8–29.25)	0.004	0.20	0.20
Complications, n (%)							
ICU admission	29 (36.3)	17 (40.5)	8 (72.7)	36 (29.8)	0.65	0.05	0.34
Mechanical ventilation	14 (17.5)	15 (35.7)	7 (63.6)	30 (24.8)	0.03	0.01	0.22
ARDS	4 (5.0)	5 (11.9)	3 (27.3)	6 (5.0)	0.31	0.07	1.00
Sepsis	41 (51.3)	28 (66.7)	7 (63.6)	6 (5.0)^b^	0.10	0.44	n/a
Mortality (all cause)	6 (7.5)	4 (9.5)	1 (9.1)	6 (5.0)	0.94	1.00	0.65
Antibiotic use (any indication)							
Any inpatient antibiotics, n (%)	58/73 (79.5)	35/38 (92.1)	9/11 (81.8)	90/109 (82.6)	0.09	1.00	0.60
Duration of inpatient use, mean days ± SD (range)	10.5 ± 13.9 (0-73)	17.5 ± 15.3 (0–72)	11.9 ± 10.0 (0–31)	10.0 ± 10.6 (0–63)	0.02	0.75	0.80
Discharged receiving oral antibiotics, n (%)	17 (23.0)^c^	17 (44.7)^d^	2 (20.0)^e^	26 (22.8)^f^	0.02	1.00	0.98

*ARDS* acute respiratory distress syndrome, *ICU* intensive care unit, *IQR* interquartile range, *LOS* length of stay, *n* number, *SD* standard deviation

^a^For continuous variables, 2-sample independent t-test or Mann-Whitney-U-test were used. For categorical variables, Mantel-Haenszel chi square or Fisher exact test were used

^b^Sepsis requires a pathogen per definition. In these six cases sepsis was exceptionally defined according to discharge papers

Missing values due to death or ongoing hospitalisation at time of analysis (number): c6; d4; e1; f7. For calculation of percentage and *p*-value missing values were excluded

### Influence of rtPCR testing on management

The effect of rtPCR analysis on antibiotic management is presented in Tables 8, 9 and 10. After exclusion of patients who received antibiotics for other indications virus detection was temporally associated with discontinuation of antibiotics in 2 of 20 adults (10%) and 6 of 14 children (43%). In patients with viral etiology, management was more frequently judged correct in children (18/27, 67%) than in adults (12/35, 34%; *p* = 0.01) after rtPCR results became available. In adults, management of viral etiology was less often judged correct compared to adults with bacterial etiology (*p* = 0.002). Among patients with clinically viral etiology, children were more frequently managed correctly (15/15, 100%) than adults (8/13, 62%; *p* = 0.03).

**Table 8:** Change of antibiotic therapy after rtPCR, adult patients with another indication for antibiotics were excluded

	*Viral*	*Bacterial*	*Mixed*	*No pathogen*	*Clinically*	*Clinically*	*p-value* ^*c*^		
	*(n = 35)*	*(n = 33)*	*(n = 6)*	*(n = 84)*	*bacterial* ^a^ *(n = 84)*	*viral* ^b^ *(n = 13)*	*Viral vs. bacterial*	*Viral vs. mixed*	*Viral vs. no pathogen*
No antibiotic treatment before and after rtPCR, n (%)	*10 (28.6)*	7 (21.2)	2 (33.3)	32 (38.1)	18 (21.4)	*8 (61.5)*	0.49	1.00	0.32
Antibiotic treatment stopped after rtPCR, n (%)	*2 (5.7)*	2 (6.1)	0 (0.0)	2 (2.4)	4 (4.8)	*0 (0.0)*	1.00	1.00	0.67
Antibiotic treatment started after rtPCR, n (%)	5 (14.3)	*4 (12.1)*	*2 (33.3)*	8 (9.5)	*7 (8.3)*	3 (23.1)	1.00	0.54	0.64
Antibiotic treatment before and after rtPCR, n (%)	18 (51.4)	*20 (60.6)*	*2 (33.3)*	42 (50.0)	*55 (65.5)*	2 (15.4)	0.45	0.71	0.89
Correct management, n (%)	*12 (34.3)*	*24 (72.7)*	*4 (66.7)*		*62 (73.8)*	*8 (61.5)*	*0.002*	0.30	n/a

*n* number; rtPCR, real-time polymerase chain reaction

^a^Cases without detection of bacteria were evaluated as clinically bacterial if they fulfilled one of the following criteria: unilobular or multilobular pulmonary infiltrate *or* CRP >100 mg/l *or* PCT >0.25 μg/l *or* antibiotic therapy before rtPCR and no bacterium detection and biomarkers indeterminate (CRP >100 mg/l and PCT ≤0.25 μg/l *or* CRP between 51 and 100 mg/l and PCT not available)

^b^Cases with a detected viral pathogen excluding those patients with a clinically bacterial co-infection (as described above)

^c^Mantel-Haenszel chi square test or Fisher exact test were used

**Table 9:** Change of antibiotic therapy after rtPCR, pediatric patients with another indication for antibiotics were excluded

	*Viral*	*Bacterial*	*Mixed*	*No pathogen*	*Clinically*	*Clinically*	*p-value* ^*c*^		
	*(n = 27)*	*(n = 4)*	*(n = 3)*	*(n = 18)*	*bacterial* ^a^ *(n = 22)*	*viral* ^b^ *(n = 15)*	*Viral vs. bacterial*	*Viral vs. mixed*	*Viral vs. no pathogen*
No antibiotic treatment before and after rtPCR, n (%)	*12 (44.4)*	1 (25.0)	0 (0.0)	6 (33.3)	6 (27.3)	*11 (73.3)*	0.87	0.40	0.46
Antibiotic treatment stopped after rtPCR, n (%)	*6 (22.2)*	0 (0.0)	0 (0.0)	1 (5.6)	2 (9.1)	*4 (26.7)*	0.80	1.00	0.27
Antibiotic treatment started after rtPCR, n (%)	1 (3.7)	*0 (0.0)*	*1 (33.3)*	3 (16.7)	*3 (13.6)*	0 (0.0)	1.00	0.39	0.34
Antibiotic treatment before and after rtPCR, n (%)	8 (29.6)	*3 (75.0)*	*2 (66.7)*	8 (44.4)	*11 (50.0)*	0 (0.0)	0.23	0.50	0.32
Correct management, n (%)	*18 (66.7)*	*3 (75.0)*	*3 (100.0)*		*14 (63.6)*	*15 (100.0)*	1.00	0.66	n/a

*n* number; rtPCR, real-time polymerase chain reaction

^a^Cases without detection of bacteria were evaluated as clinically bacterial if they fulfilled one of the following criteria: unilobular or multilobular pulmonary infiltrate *or* CRP >100 mg/l *or* PCT >0.25 μg/l *or* antibiotic therapy before rtPCR and no bacterium detection and biomarkers indeterminate (CRP >100 mg/l and PCT ≤0.25 μg/l *or* CRP between 51 and 100 mg/l and PCT not available)

^b^Cases with a detected viral pathogen excluding those patients with a clinically bacterial co-infection (as described above)

^c^Mantel-Haenszel chi square test or Fisher exact test were used

**Table 10:** Change of antibiotic therapy after rtPCR, all patients with another indication for antibiotics were excluded

	*Viral*	*Bacterial*	*Mixed*	*No pathogen*	*Clinically*	*Clinically*	*p-value* ^*c*^		
	*(n = 62)*	*(n = 37)*	*(n = 9)*	*(n = 102)*	*bacterial* ^a^ *(n = 106)*	*viral* ^b^ *(n = 28)*	*Viral vs. bacterial*	*Viral vs. mixed*	*Viral vs. no pathogen*
No antibiotic treatment before and after rtPCR, n (%)	*22 (35.5)*	8 (21.6)	2 (22.2)	38 (37.3)	24 (22.6)	*19 (67.9)*	0.15	0.71	0.82
Antibiotic treatment stopped after rtPCR, n (%)	*8 (12.9)*	2 (5.4)	0 (0.0)	3 (2.9)	6 (5.7)	*4 (14.3)*	0.40	0.64	0.03
Antibiotic treatment started after rtPCR, n (%)	6 (9.7)	*4 (10.8)*	*3 (33.3)*	11 (10.8)	*10 (9.4)*	3 (10.7)	1.00	0.16	0.82
Antibiotic treatment before and after rtPCR, n (%)	26 (41.9)	*23 (62.2)*	*4 (44.4)*	50 (49.0)	*66 (62.3)*	2 (7.1)	0.05	1.00	0.38
Correct management, n (%)	*30 (48.4)*	*27 (73.0)*	*7 (77.7)*		*76 (71.7)*	*23 (82.1)*	0.02	0.19	n/a

*n* number; rtPCR, real-time polymerase chain reaction

^a^Cases without detection of bacteria were evaluated as clinically bacterial if they fulfilled one of the following criteria: unilobular or multilobular pulmonary infiltrate *or* CRP >100 mg/l *or* PCT >0.25 μg/l *or* antibiotic therapy before rtPCR and no bacterium detection and biomarkers indeterminate (CRP >100 mg/l and PCT ≤0.25 μg/l *or* CRP between 51 and 100 mg/l and PCT not available)

^b^Cases with a detected viral pathogen excluding those patients with a clinically bacterial co-infection (as described above)

^c^Mantel-Haenszel chi square test or Fisher exact test were used

Eight adults and one child received oseltamivir. In four of five patients with proven influenza A virus infection, the antiviral medication was prescribed in response to the positive rtPCR analysis. In another child, oseltamivir was commenced after the third successive detection of influenza A virus within a month and stopped after 1 day. In two other patients, antiviral therapy was started or continued despite an rtPCR analysis negative for influenza and positive for other respiratory viruses. In another patient with identification of only bacterial pathogens, oseltamivir was stopped after negative rtPCR results were available. In an additional case with detection of bacteria only, antiviral medication was started 3 days after rtPCR analysis and it was stopped again on the next day.

### Outcomes depending on etiology

Hospital length of stay (LOS) was longer in patients with bacterial etiology compared to patients with a viral etiology. For children, LOS did not significantly vary in the different groups between viral and bacterial etiologies (Tables 5, 6 and 7). Patients with a mixed infection were more frequently admitted to the intensive care unit (ICU) and mechanically ventilated compared to patients with a viral etiology (*p* = 0.05; *p* = 0.005; respectively). If children and adults were analyzed separately, the differences within the adult population were not significant; in children, more patients with a mixed infection were mechanically ventilated (*p* = 0.03). All-cause and RTI-associated mortality was comparable between pathogen groups.

In patients with viral etiology there was no difference in mortality between those who discontinued antibiotics compared to those who did not (1/8 [13%] vs. 3/26 [12%]; *p* = 1.00). LOS was shorter in inpatients who discontinued antibiotics (median days 5 [IQR 3–11.75] vs. 10.5 [IQR 6–19.25]; *p* = 0.05). Patients in whom antibiotics were continued despite a viral detection were as frequently admitted to ICU (11/26 [42%] vs. 5/8 [63%]; *p* = 0.55) or mechanically ventilated (6/26 [23%] vs. 1/8 [13%]; *p* = 0.93) as patients in whom antibiotics were discontinued. The proportion of patients with sepsis was comparable (16/26 [62%] vs. 6/8 [75%]; *p* = 0.80). These results were similar if adults and children were analyzed separately.

### Radiological findings depending on etiology

Normal CXR findings were significantly more frequent in patients with viral infection compared to bacterial infections (30% vs. 9%; *p* = 0.03). Multilobar infiltrates and pleural effusion on CXR were observed less often among subjects with viral infection (Tables 11, 12 and 13).

**Table 11:** Radiological findings for adult patients

	*Viral*	*Bacterial*	*Mixed*	*No pathogen*		*p-value* ^*a*^	
	*(n = 40)*	*(n = 36)*	*(n = 6)*	*(n = 100)*	*Viral vs. bacterial*	*Viral vs. mixed*	*Viral vs. no pathogen*
X-ray, (n)	33	26	5	81			
Unilobar infiltrate, n (%)	9 (27.3)	5 (19.2)	0 (0.0)	14 (17.3)	0.48	0.47	0.23
Multilobar infiltrates, n (%)	6 (18.2)	10 (38.5)	2 (40.0)	30 (37.0)	0.09	0.56	0.05
Interstitial infiltrates, n (%)	3 (9.1)	7 (26.9)	1 (20.0)	17 (21.0)	0.14	0.89	0.13
Pleural effusion, n (%)	5 (15.2)	11 (42.3)	0 (0.0)	24 (29.6)	0.02	0.95	0.11
Normal, n (%)	8 (24.2)	2 (7.7)	1 (20.0)	14 (17.3)	0.18	1.00	0.40
Computer tomography, (n)	23	29	5	71			
Unilobar infiltrate, n (%)	5 (21.7)	4 (13.8)	0 (0.0)	7 (9.9)	0.70	0.69	0.26
Multilobar infiltrates, n (%)	11 (47.8)	16 (55.2)	4 (80.0)	32 (45.1)	0.60	0.42	0.82
Ground glass opacity, n (%)	4 (17.4)	6 (20.7)	2 (40.0)	29 (40.8)	1.00	0.57	0.04
Pleural effusion, n (%)	5 (21.7)	13 (44.8)	1 (20.0)	25 (35.2)	0.09	1.00	0.23
Normal, n (%)	2 (8.7)	0 (0.0)	0 (0.0)	0 (0.0)	0.38	1.00	0.12

*n* number. Multiple findings were counted individually

^a^Mantel-Haenszel chi square test or Fisher exact test were used

**Table 12:** Radiological findings for pediatric patients

	*Viral*	*Bacterial*	*Mixed*	*No pathogen*		*p-value* ^*a*^	
	*(n = 40)*	*(n = 6)*	*(n = 5)*	*(n = 21)*	*Viral vs. bacterial*	*Viral vs. mixed*	*Viral vs. no pathogen*
X-ray, (n)	28	6	5	16			
Unilobar infiltrate, n (%)	6 (21.4)	0 (0.0)	1 (20.0)	2 (12.5)	0.56	1.00	0.76
Multilobar infiltrates, n (%)	6 (21.4)	2 (33.3)	4 (80.0)	6 (37.5)	0.88	0.04	0.42
Interstitial infiltrates, n (%)	2 (7.1)	1 (16.7)	1 (20.0)	2 (12.5)	0.91	0.80	0.93
Pleural effusion, n (%)	4 (14.3)	2 (33.3)	1 (20.0)	2 (12.5)	0.56	1.00	1.00
Normal, n (%)	10 (35.7)	1 (16.7)	0 (0.0)	3 (18.8)	0.70	0.28	0.40
Computer tomography, (n)	5	1	2	3			
Unilobar infiltrate, n (%)	2 (40.0)	0 (0.0)	0 (0.0)	0 (0.0)	1.00	0.95	0.71
Multilobar infiltrates, n (%)	2 (40.0)	1 (100.0)	2 (100.0)	3 (100.0)	1.00	0.57	0.36
Ground glass opacity, n (%)	0 (0.0)	0 (0.0)	1 (50.0)	0 (0.0)	n/a	0.57	n/a
Pleural effusion, n (%)	2 (40.0)	1 (100.0)	1 (50.0)	2 (66.7)	1.00	1.00	1.00
Normal, n (%)	1 (20.0)	0 (0.0)	0 (0.0)	0 (0.0)	1.00	1.00	1.00

*n* number. Multiple findings were counted individually

^a^Mantel-Haenszel chi square test or Fisher exact test were used

**Table 13:** Radiological findings for all patients

	*Viral*	*Bacterial*	*Mixed*	*No pathogen*		*p-value* ^*a*^	
	*(n = 80)*	*(n = 42)*	*(n = 11)*	*(n = 121)*	*Viral vs. bacterial*	*Viral vs. mixed*	*Viral vs. no pathogen*
X-ray, (n)	61	32	10	97			
Unilobar infiltrate, n (%)	15 (24.6)	5 (15.6)	1 (10.0)	16 (16.5)	0.32	0.57	0.21
Multilobar infiltrates, n (%)	12 (19.7)	12 (37.5)	6 (60.0)	36 (37.1)	0.06	0.03	0.02
Interstitial infiltrates, n (%)	5 (8.2)	8 (25.0)	2 (20.0)	19 (19.6)	0.06	0.51	0.05
Pleural effusion, n (%)	9 (14.8)	13 (40.6)	1 (10.0)	26 (26.8)	0.01	1.00	0.08
Normal, n (%)	18 (29.5)	3 (9.4)	1 (10.0)	17 (17.5)	0.03	0.37	0.08
Computer tomography, (n)	28	30	7	74			
Unilobar infiltrate, n (%)	7 (25.0)	4 (13.3)	0 (0.0)	7 (9.5)	0.26	0.35	0.10
Multilobar infiltrates, n (%)	13 (46.4)	17 (56.7)	6 (85.7)	35 (47.3)	0.44	0.14	0.94
Ground glass opacity, n (%)	4 (14.3)	6 (20.0)	3 (42.9)	29 (39.2)	0.82	0.25	0.02
Pleural effusion, n (%)	7 (25.0)	14 (46.7)	2 (28.6)	27 (36.5)	0.09	1.00	0.28

*n* number. Multiple findings were counted individually

^a^Mantel-Haenszel chi square test or Fisher exact test were used

### Predictors of viral etiology

In multivariate logistic regression (Tables 14 and 15), the absence of pleural effusion in adults was associated with detection of respiratory viruses (odds ratio [OR] 0.31, 95% confidence interval [CI] 0.12–0.80). For children, the lack of multilobar infiltrates was a significant predictor of respiratory virus detection (OR 0.22, 95% CI 0.06–0.81).

**Table 14:** Prediction of respiratory virus detection for adult patients

	*Univariable analysis*		*Multivariable analysis*	
Predictor	*p-value*	*OR (95% CI)*	*p-value*	*OR (95% CI)*
Neutropenia	0.01	3.46 (1.32–9.07)	0.27	2.11 (0.56–7.89)
Hematologic malignancy	0.04	2.25 (1.02–4.97)	0.59	1.35 (0.45–4.01)
Pleural effusion	0.02	0.36 (0.15–0.86)	0.02	0.31 (0.12–0.80)
Platelets (minimum), G/l	0.02	1.00 (0.99–1.00)	0.20	1.00 (0.99–1.00)

**Table 15:** Prediction of respiratory virus detection for pediatric patients

	*Univariable analysis*		*Multivariable analysis*	
Predictor	*p-value*	*OR (95% CI)*	*p-value*	*OR (95% CI)*
Neutropenia	0.06	7.75 (0.92–65.66)	0.10	7.44 (0.69–80.42)
Multilobular infiltrates	0.03	0.31 (0.11–0.91)	0.02	0.22 (0.06–0.81)
White blood cells (maximum), G/l	0.02	0.94 (0.89–0.99)	0.10	0.95 (0.90–1.01)

*OR* odds ratio, *CI* confidence interval

## Discussion

There are three main findings in this retrospective cohort study of the impact of viral multiplex rtPCR. First, the majority of patients with viral RTI received antibiotics and antibiotics were discontinued after viral detection in only a minority of patients. Second, when biomarkers, radiologic presentations and antibiotic pre-treatment were taken into account and categories of clinically bacterial and clinically viral infections were created (which more closely reflect clinical decision making), the multiplex rtPCR showed a greater impact and considerably improved correct management of clinically viral infections, from 67 to 100% among children and from 34 to 62% among adults. Third, the impact of rtPCR testing seemed to be more accentuated in children than in adults. More children than adults had an appropriate discontinuation of antibiotics, and the overall management of viral infections was superior in children compared to adults. Importantly, but with the caveat of small numbers, there was no evidence that outcome was worse in those with viral etiology who discontinued antibiotics compared to those who did not.

Several previous studies analyzed the impact of rapid availability of rtPCR results on antibiotic use. In a randomized controlled trial (RCT) of 107 adults with lower RTI, antibiotics were partially or totally discontinued in 6 (11%) of 55 patients for whom rtPCR results were available, albeit without overall reduction in antibiotic treatment duration [7]. In a controlled clinical trial enrolling 583 children with acute RTI, Wishaupt et al. [4] evaluated the diagnostic yield and effect of rapid communication of rtPCR results (within 12–36 h vs. 4 weeks after testing) and failed to show a significant influence on the duration of antibiotic treatment. In contrast, Brittain-Long et al. [20] demonstrated in a RCT with 406 adults that patients randomized to rapid rtPCR results received antibiotics less frequently for acute RTI in a primary care setting during their initial visit (4.5% vs. 12.3%; *p* = 0.01). However, at the 10-day follow-up the prescription rates were similar again (13.9% vs. 17.2%; *p* = 0.36) [20]. Contrary to these findings, a retrospective pre-post study of 1136 children showed that the introduction of an expanded multiplex rtPCR had a shorter turnaround time and decreased the duration of antibiotic use (2.8 vs. 3.2 days; *p* = 0.003) without reducing the proportion of antibiotic prescriptions [26].

As in most previous studies, typical respiratory bacteria were not included in the test panel. Due to this technical limitation, missing a treatable pathogen is of concern in light of potential bacterial-viral co-infections [3, 7–13, 16–19]. It was shown that clinical suspicion of a bacterial super-infection was one reason for physicians to not stop antibiotics in rtPCR-positive patients [3]. We tried to partially overcome this by creating categories of clinically bacterial and clinically viral infections. This definition, aided by providing a biomarker criterion, accounted for the possibility of a viral infection in the setting of bacterial carriage. One of the major differences and advantages of the current study compared to previous publications is that it not only assessed the impact of the rtPCR results, which themselves had a limited impact on appropriate therapy, but it also integrated other ‘real-world’ clinical and radiologic parameters into the decision process.

Advanced molecular diagnostic tests have to be interpreted in the context of available clinical and diagnostic information in order to improve clinical management. The results confirmed the important role of clinical judgement for appropriate antibiotic prescriptions, with rtPCR providing additional information rather than being solely responsible for treatment decisions.

Quantification of genomic viral load might improve specificity of virus detection, with higher organism burden being associated with higher risk of complications and severe disease in adults and children [27, 28]. Unfortunately, quantitative results were not available with the applied assay. Similarly, optimal timing of molecular testing in relation to symptom onset and inclusion of an ever-expanding number of respiratory viruses might be important to further increase sensitivity [27]. However, to the authors’ knowledge, it has not yet been studied whether either of these two factors would improve clinical management.

A pathogen was identified in 140 (55%) patients in this study, in accordance with detection rates in other studies ranging from 38 to 82% [4, 12, 17, 19, 20, 22, 29–35]. A mixed bacterial-viral etiology was found in 11 (8%) patients. Previously described rates varied between 2 and 23% [17, 19, 22, 29–35]. Some studies suggested that mixed bacterial-viral infections result in more severe clinical diseases (as measured by the CURB-65 score or the pneumonia severity index) [17, 34], a higher rate of mechanical ventilation, longer duration of ICU care [36, 37], longer hospital stays [22, 36], or higher mortality [37, 38], while other studies did not [29, 39, 40]. In this study, mixed bacterial-viral infections were associated with a higher rate of ICU admission and mechanical ventilation compared to pure viral (*p* = 0.05; *p* = 0.005) and pure bacterial (*p* = 0.06; *p* = 0.19) infections if all patients were considered. However, these results should not be viewed as representative of the etiology of lower RTI in East Switzerland as these hospitals presented a preselected group of patients.

In agreement with the findings of some studies [20, 22, 34] and in contrast to others [7, 29, 39], this study failed to identify specific predictors of viral detection in multivariate logistic regression. Advancing age was previously described as more common in viral infections [19, 22, 29, 35] but the evidence is inconclusive [31, 32]. In this study, younger age was a significant predictor of virus detection in univariable logistic regression if all patients were considered, but not in stratified analyses in children and adults. The mean age in the group with respiratory viral infections was lower than in the remaining patients (29 vs. 47 years; *p* < 0.001) but the differences were not significant if stratified for children and adults.

There are some limitations to the study. First, the change of anti-infective management was retrospectively matched with the date of the rtPCR analysis. Exact time specifications were not available, leaving room for potential inaccuracies. It is not known whether and to what extent these or other factors contributed to the clinical decisions in starting, stopping, or continuing antibiotic therapy. Due to the retrospective nature of the study, it was not possible to consider the clinical presentation in the analysis. Therefore, it was difficult to reproduce the decision-making process and the primary indication for antibiotic therapy. This is an important limitation because clinical judgement remains essential concerning the use of antibiotics [41]. Furthermore, data on the consequences of rtPCR results on antibiotic treatment are difficult to obtain in the ambulatory setting, which explains the relatively small number of enrolled outpatients. Prospective studies or ideally RCTs will be necessary to confirm the findings.

Second, creating categories of clinically bacterial and clinically viral etiology by means of biomarker, radiologic presentations and antibiotic pre-treatment was done arbitrarily to reflect real-life decision-making. These theoretical reflections were important to better understand the findings; however, and as noted above, retrospectively the actual decision-making process remained unclear.

Third, the study included patients with upper and lower respiratory tract infections and did not exclude patients with antibiotic pre-treatment or significant underlying pathologic conditions, which may have biased the results but reflects clinical routine. Not having algorithms in place when to perform rtPCR testing or how to apply the results likewise mirrors the real-world scenario.

Fourth, the study’s multiplex rtPCR only included respiratory viruses. Newer generation assays additionally cover atypical and typical bacteria and increase the detection of potential respiratory pathogens, albeit with yet unresolved specificity issues as these typical bacterial pathogens might also represent carriage in the absence of disease [42].

Fifth, the prevalence of RSV was underestimated in this study. In the population of hospitalized children, rapid detection tests for RSV were performed initially and, if positive, no additional rtPCR was performed. Because the study included only patients in whom an rtPCR assay for respiratory viruses was performed, the number of RSV infections was lower than expected from epidemiological data.

## Conclusion

This study reveals the real-life impact of viral multiplex rtPCR in both children and adults, which was more limited in adults but improved when results were seen in the context of biomarkers, radiology, and antibiotic pre-treatment. As substantial reduction of unnecessary antibiotic prescriptions seems possible, it will be necessary to develop more structured management algorithms incorporating molecular diagnostics including bacterial pathogens, which need to be prospectively tested in their efficacy and safety in RTI.

## Abbreviations

CRP: C-reactive protein
CT: Computer tomography
CXR: Chest radiograph
ICU: Intensive care unit
IQR: Interquartile range
LOS: Length of stay
PCT: Procalcitonin
RCT: Randomized controlled trial
RSV: Respiratory syncytial virus
RTI: Respiratory tract infection
rtPCR: Real-time polymerase chain reaction
SIRS: Systemic inflammatory response syndrome
WBC: White blood cells count
